# Determinants of coexistence of stunting, wasting, and underweight among children under five years in the Gambia; evidence from 2019/20 Gambian demographic health survey: application of multivariate binary logistic regression model

**DOI:** 10.1186/s12889-022-14000-3

**Published:** 2022-08-26

**Authors:** Abebew Aklog Asmare, Yitateku Adugna Agmas

**Affiliations:** 1 Department of Statistics, Mekdela Amba University, P.O. Box: 32, Tuluawlyia, Ethiopia; 2 Department of Rural Development and Agricultural Extension, Mekdela Amba University, P.O. Box: 32, Tuluawlyia, Ethiopia

**Keywords:** Gambia, Multivariate binary logistic regression, Under-five children, Undernutrition

## Abstract

**Background:**

Malnutrition happens when there are insufficient amounts of nutrients and energy consumed improperly. Included are both undernutrition and overnutrition. This study is aimed to evaluate the relationship among undernutrition indicators of stunting, underweight, and wasting among those under 5 years given other predictors.

**Methods:**

The data were obtained from the measure of DHS program. A total of 2399 under-five children were involved in this study. A multivariate binary logistic regression model is used to assess the association between stunting, wasting, and being underweight given the effect of other predictors.

**Results:**

Of the 2399 under-five children considered in this study, 13.5, 18.7, and 5.9% of them suffered from stunting, underweight, and wasting, respectively. The majority of children (40.1%) were obtained from the Brikama local government area of Gambia; more than half of the children (52.9%) were male, and 63.3% of children lived in urban areas. The association between stunting and underweight, underweight and wasting, and stunting and wasting was measured by the odds ratio (OR) of 15.87, 46.34, and 1.75, respectively, given the other predictors. The estimated odds ratio for children who had an average birth size to become stunted, underweight, and wasted were 0.965, 0.885, and 0.989 times the estimated odds ratio of children who had a small birth size, respectively.

**Conclusion:**

The prevalence of stunting and wasting for under-five children in Gambia was lower than the world prevalence, but the prevalence of being underweight was higher. Children who are underweight have a significant association with both stunting and wasting. The age of the child, the child’s anemia level, and the birth type of the child are the common important determinants of stunting and underweight. The small birth size of a child was highly associated with a higher risk of stunting, underweight, and wasting among under five-year-olds.

## Background

Malnutrition happens when there are insufficient amounts of nutrients and energy consumed improperly. Included are both undernutrition and overnutrition [1, 2]. Under-five children who are malnourished experience nutritional deficits as a result of the interaction between illness and poor nutrition [3, 4]. Stunting, wasting, and underweight are disorders connected to undernutrition. It is impacted by a lack of health services; a terrible environment; insufficient financial, physical, and social capital; inadequate food consumption; infection; poor food access; bad care and feeding habits; and poor care feeding habits [5–9]. It is also one of the leading causes of illness and death among under-five children in developing countries like Gambia [10]. Furthermore, malnutrition remains the leading cause of disease [11, 12], with long-term consequences such as impaired cognitive development, insufficient growth, and poor academic performance [13]. Under-five children are the most vulnerable to undernutrition in developing countries due to inadequate nutritional intake, lack of appropriate care, and unbalanced food distribution within the household [14]. The occurrence of undernutrition symptoms in children is a close indicator of overall nutrition and nutrition security conditions in low and middle-income countries [15].

Children who receive nourishing food are more likely to survive and thrive. When children are well-nourished, they develop, learn, play, participate, and contribute; however, when they are malnourished, they do not attain their full potential, which has an impact on the world’s children, nations, and societies [16]. The three widely accepted indicators of malnutrition in under-five children are stunting, wasting, and underweight [17]. Stunting and wasting signify, respectively, chronic and acute malnutrition. Undernutrition that is both acute (wasting) and chronic (stunting) may be indicated by underweight [17]. Children may experience multiple forms of malnutrition at the same time [17]. A baby who is simply too short for his or her age (low height-for-age) has been referred to as stunting [18]. Wasting is also defined as a baby who is simply too small for his or her height (low weight-for-height). Underweight refers to a baby who is simply too small for his or her age (low weight-for-age), which means that the weight for age is less than negative two standard deviations (SD) below the WHO Child Growth Standards median [18, 19].

Malnutrition has various causes, which include illnesses, inadequate diets, and environmental and socioeconomic characteristics [20]. The age of the child in a month [20–23], gender of the child [24, 25], birth size of the child [26, 27], birth order [4, 28], maternal education [28, 29], mother’s body mass index [24, 29], household wealth index [4, 21, 30], source of drinking water [22, 30], family size [24], region [22], residence [24], religion [31], ethnicity [32], sex of household head [30], husband education level [4, 30], breastfeeding status [32, 33], sex of children [34, 35], diarrhea [32, 36], fever and cough [31, 32] in the last 2 weeks prior to the survey, birth type of children [22, 28], number of under-five children [27], maternal anemia [9] and child anemia [22] were the determinants of children’s nutritional status in Gambia which have been identified.

According to the United Nations Children’s Fund [16], undernutrition is responsible for nearly half of all deaths among children under the age of five worldwide in 2020; 22% of children under the age of five were stunted, 12.6% were underweight, and 6.7% were wasted [16]. In the same year, approximately 149.2 million under-five children were affected by stunting. Asia was responsible for 30.7% of all stunted children [16]. Wasting will have contributed to the extinction of 45.4 million children under the age of five. More than two-thirds of all wasted children are found in Asia, with Africa accounting for more than a quarter [16]. However, a closer look at the distribution of stunting in the African region reveals that Eastern Africa (32.6%) has a higher incidence of stunting than Western Africa (30.9%), Central Africa (36.8%), Northern Africa (21.4%), and Southern Africa (23.3%) [16]. While Western Africa has a higher rate of wasting than the rest of Africa, Southern Africa (3.2%), Central Africa (6.2%), Northern Africa (6.6%), and Eastern Africa (5.2%) have lower rates [16].

Gambia, which stretches 400 km from the Atlantic Ocean to the east along the Gambia River, is one of the poorest Western African countries in the world, ranking 165 on the UNDP Human Development Index [20]. The newborn mortality rate is 42 deaths per 1000 live births [37]. Underweight, wasting, and stunting affected 12, 5, and 18% of children under the age of five, respectively [37].

To the best of the researchers’ knowledge, no study has been conducted on the determinants of the coexistence of undernutrition indicators in Gambia. In Nepal, Ethiopia, Uganda, Bangladesh, East Africa, Cameron, Ghana, Sub-Sahara Africa, Gambia, Tanzania, and Mozambique [4, 10, 20, 22, 27, 28, 33–35, 37–39], stunting, underweight, and wasting have all been extensively studied. However, little attention has been paid to their association, and literature is scarce, particularly in Gambia, so we will conduct a separate analysis of stunting, underweight, and wasting in children under the age of five, as previously done by studies [4, 10, 20, 22, 27, 28, 33–35, 37–39], using ordinal or binary logistic regression. However, when using binary or ordinal logistic regression, the association between stunting, underweight, and wasting is ignored. To do so, we consider the correlation between the indicators of undernutrition and, as a result, assess the special effects of other predictors. As a result, multivariate logistic regression may be a better option. This statistical model is used to simulate two or more binary outcome variables at the same time and assess their relationship in relation to other predictors [40, 41]. It meets the criteria for modeling marginal likelihood as a function of explanatory variables. At the same time, the model examines the relationship between stunting, underweight, and wasting in children under the age of five.

Though stunting and wasting are commonly presented as two distinct types of undernutrition that necessitate different interventions for prevention and/or treatment, they are closely related and frequently occur together in the same people and frequently in identical children. Stunting and wasting are associated with an increased risk of death, particularly when both occur in the same child [42]. A study was done in India, Malawi, and Ethiopia [43–45] looked at the link between stunting, underweight, and wasting. However, the effect of other predictors associated with stunting, underweight, and wasting was not taken into account when measuring the relationship. Thus, using multivariate binary logistic regression analysis, this study aims to assess the relationship between stunting, underweight, and wasting in under-five children in relation to other predictors.

During this study, two hypotheses were tested. The first hypothesis asserts that there is no relationship between the three undernutrition indicators among children under the age of five, while the second asserts that there is no relationship between predictors and undernutrition indicators. As a result, a better understanding of the relationship between stunting, underweight, and wasting will aid the concerned body in developing targeted interventions to improve child health and survival. As a result, the current study will benefit policymakers at both the governmental and personal levels by providing evidence on which interventions and policy actions are frequently formulated and implemented for children under the age of five.

## Method

### Data source and sampling method

We used data from the 2019/20 Gambian Demographic and Health Surveys (GDHS). These GDHSs are nationally representative cross-sectional surveys conducted in eight local area governments every 5 years. This survey employed stratified two-stage cluster sampling. Each local government was stratified by dividing it into urban and rural areas. So far, 14 sampling strata have been created. A total of 281 Enumeration Areas (EAs) were chosen at random in proportion to their size in the 2019/20 GDHS. In the second phase, 25 households were chosen on average per enumeration area [37].

After approval was granted via an online request stating the purpose of this study, the data was retrieved from the Measure DHS website https://dhsprogram.com/Data/terms-of-use.cfm. The dependent and independent variables in this study were extracted from the Kid Record (KR file) data set. This study used a weighted total sample of 2399 children under the age of five. The entire sampling technique was described in detail in the comprehensive GDHS report [37].

### Inclusion/exclusion criteria

The inclusion criteria were children under the age of five who completed relevant forms containing personal information and clinical signs. As a result, children who had not completed all related information or who were older than or equal to 5 years old were excluded.

### Study variables and measurements

#### Dependent variables

Height-for-age (stunting), weight-for-height (wasting), and weight-for-age (underweight) were calculated using a standardized score (z-score) based on WHO 2006 child growth standards [46]. The Z-score for the *i*^*th*^ child (*Z*_*i*_) is defined as ${Z}_i=\frac{AI_i-\mu }{\sigma }$, where *AI*_*i*_, *μ and σ* is stunting, underweight, and wasting of the *i*^*th*^ child, median and standard deviation, respectively. After the *Z*_*i*_ for each child is calculated, Stunting is defined as a baby being too short for his or her age (low height-for-age). Wasting has also been defined as a baby who is simply too small for his or her height (low weight-for-height). Similarly, an underweight baby is one who is simply too small for his or her age (low weight-for-age). The response variables were recoded into dichotomies as follows:

$$stunted\ \left(0= No\ if\ HAZ\ge -2\ and\ 1= Yes\ if\ HAZ<-2\right),$$

$$wasted\ \left(0= No\ if\ WHZ\ge -2\ and\ 1= Yes\ if\ WHZ<-2\right), and$$

$$underweight\ \left(0= No\ if\ WAZ\ge -2\ and\ 1= Yes\ if\ WAZ<-2\right).$$

#### Independent Variables

The independent variables included in this study were region, types of place of residence, highest education level of mother, source of drinking water, religion, number of household members, sex of household head, ethnicity, age of respondent at first birth, husband’s education level, breast feeding, wealth index of household, body mass index of mother, sex of a child, child age in a month, diarrhea, fever, cough, size of children at birth, whether a child is a twin, birth order of a child, number of under-five children in the household, mother anemia level, and child anemia level. The above listed independent variables which are correlated to the three undernutrition indicators were presented in (Table 2), and the parameters in Table 2 were collected using face-to-face interviews of mothers or care-givers.

#### Ethics approval and consent to participate

Hence, permission to get access to the data was obtained from the measure DHS program online request from https://dhsprogram.com/Data/terms-of-use.cfm website, and the data used was publicly available with no personal identifier.

### Data management

The information was obtained from the KID Record (KR file) data sets. To restore the survey’s representativeness and obtain reliable statistical estimates, the data were weighted using sampling weight for probability sampling and non-response before any statistical analysis was performed. It was first imported and managed using SPSS version 26 software. Lastly, the analysis was executed by R software version 4.0.5 using the VGAM package [47]. VGAM was accustomed to delivering functions designed for fitting vector generalized linear and additive models.

### Statistical analysis

#### Descriptive statistics

Descriptive statistics are a collection of brief descriptive figures that summarize a given data set, which can be a sample representation. Descriptive statistics were used in this study to determine the frequency and percentage of both outcome variables and independent variables.

#### Inferential Statistics

Based on Pearson’s chi-square statistic, the bivariate analysis provides preliminary insight into the association/relationship between all independent variables included in this study and the dependent variable. High Pearson chi-square values for a given independent variable indicate that there is a strong association between each of the given independent variables and the dependent variables while controlling for the effect of other factors. The chi-square value and *p* value at the 0.05 level of significance were used to make the decision. A multivariate binary logistic regression model is used in this study to estimate the effect of independent variables on the dichotomous outcomes of stunting, underweight, and wasting in children under the age of five. A multivariate binary logistic regression model is a statistical model for estimating the effect of predictors on binary outcome variables. In the multivariate binary logistic regression model, the research hypothesis states that the success probabilities are dependent on the independent variables. For large samples, the significance of each independent variable is determined using the Wald test statistic, which has a standard normal distribution. The best method for measuring the relationship between categorical variables in the logistic regression model was the odds ratio, which is the proportion of odds [48].

#### The goodness of fit test

An examination of the model’s adequacy or goodness of fit is required prior to fitting the model. This could be perceived through the predictive power of the evaluated model. The concordance proportion is commonly used to measure or detect predictive power in multivariate logistic regression models. The concordance value estimates the likelihood that predictions and outcomes are concordant, that is, whether the expected response matches the observed response [48]. As a result, in this study, the concordance proportion was calculated to determine how well the predicted model approximates the data.

## Result

### General characteristics of the study population

#### Characteristics of the outcome variables

This study included a weighted total of 2399 under-five children, with 13.5% (324), 18.7% (449), and 5.9% (142) suffering from stunting, underweight, and wasting, respectively (Table 1).

**Table 1:** Frequency and percentage distribution of the outcome variables

Outcome variable	Categories (codes)	Frequency (%)
Stunting	No (0)	2075 (86.5)
	Yes (1)	324 (13.5)
Underweight	No (0)	1950 (81.3)
	Yes (1)	449 (18.7)
Wasting	No (0)	2257 (94.1)
	Yes (1)	142 (5.9)

### Characteristics of independent variables

The result of Table 2 revealed that the majority of children under-5 years (40.1%) were obtained from the Brikama local government area of Gambia, while 63.3% of households accessed improved drinking water. 24.0% of the children were aged between 12 months and 23 months. More than half of the children (52.9%) were male, and 63.3% of the children lived in urban areas. Most of the households (62.9%) have 10 or more family members, and more than half (55.5%) of the households have three or more under-five children. The highest proportion of children (19.0%) was from the Fula/Tukulur/Lorobo ethnicity, while the lowest (0.5%) was obtained from the Manjago ethnicity. More than 23% (23.7%) of households have the poorest wealth index. Two weeks before the survey, 19.7, 17.6, and 15.6% of children had diarrhea, coughs, and fever, respectively. Almost half (46.3%) of children under 5 years old were anemic.

**Table 2:** Frequency and percentage distribution of independent variables

Variables	Categories (code)	Weighted frequency (%)
Region	Banjul(0)	26 (1.1)
	Kanifing(1)	386 (16.1)
	Brikama(2)	961 (40.1)
	Mansakanko(3)	121 (5.0)
	Kerewan(4)	277 (11.6)
	Kuntaur (5)	145 (6.0)
	Janjanbureh(6)	176 (7.3)
	Basse (7)	308 (12.8)
Type of place of residence	Urban (0)	1518 (63.3)
	Rural (1)	881 (36.7)
Highest educational level of mother	No education (0)	1139 (47.5)
	Primary education(1)	424 (17.7)
	Secondary and above(2)	836 (34.8)
Source of drinking water	Unimproved(0)	175 (7.3)
	Improved (1)	2225 (92.7)
Religion	Islam (0)	2375 (99.0)
	Christianity(1)	24 (1.0)
Number of household member	Small (1–4)	145 (6.0)
	Medium (5–9)	745 (31.1)
	Large (10 and more)	1509 (62.9)
Sex of household head	Male(0)	2037 (84.9
	Female (1)	362 (15.1)
Ethnicity	Mandinka /Jahanka(0)	820 (34.2)
	Wollof (1)	326 (13.6)
	Jola/karoninka (2)	214 (8.9)
	Fula/Tukulur/Lorobo (3)	455 (19.0)
	Serere (4)	61 (2.5)
	Serahuleh (5)	201 (8.4)
	Creole/Aku Marabout (6)	4 (0.1)
	Manjago (7)	12 (0.5)
	Bambara (8)	25 (1.0)
	Other (9)	12 (0.5)
	Non-Gambian (10)	269 (11.2)
Age of respondent at 1st birth	Less than 20	1266 (52.8)
	20 to 34	1133 (47.2)
	35 and more	1 (0.0)
Anemia level of mother	Not anemic (0)	1264 (52.7)
	Anemic (1)	1135 (47.3)
Anemia level of children	Not anemic (0)	1289 (53.7)
	Anemic (1)	1111 (46.3)
Number of under five children in the household	Only one (0)	385 (16.1)
	2 children (1)	683 (28.5)
	3 and more (2)	1331 (55.5)
Husbands educational level	No education (0)	1311 (54.6)
	Primary education (1)	517 (21.6)
	Secondary and above (2)	571 (23.8)
Breastfeeding	No (0)	1069 (44.5)
	Yes (1)	1331 (55.5)
Wealth index of the household	Poorest (0)	569 (23.7)
	Poorer (1)	505 (21.0)
	Middle (2)	508 (21.2)
	Richer (3)	406 (16.9)
	Richest (4)	412 (17.2)
Body mass index	Thin (0)	206 (8.6)
	Normal (1)	1241 (51.7)
	Overweight (2)	952 (39.7)
Sex of child	Male (0)	1269 (52.9)
	Female (1)	1130 (47.1)
Child age in month	0–11 months (0)	285 (11.9)
	12–23 months (1)	575 (24.0)
	24–35 months(2)	530 (22.1)
	36–47 months (3)	553 (23.1)
	48–59 months (4)	457 (19.0)
Diarrhea	No (0)	1927 (80.3)
	Yes (1)	472 (19.7)
Fever	No (0)	2025 (84.4)
	Yes (1)	374 (15.6)
Cough	No (0)	1976 (82.4)
	Yes (0)	423 (17.6)
Birth size of children	Small (0)	349 (14.6)
	Average(1)	1030 (42.9)
	Large (2)	1020 (42.5)
Birth type of children	Single birth (0)	2297 (98.7)
	Multiple birth (1)	102 (4.3)
Birth order of children	First (0)	367 (15.3)
	Two to three (1)	858 (35.8)
	Four to five (2)	603 (25.1)
	6 and more (3)	571 (23.8)

As shown in Table 3, children were infected by more than one of the three child undernutrition indicators, necessitating further investigation into the total number of undernourished children. For example, 86 children had both wasting and underweight; see the results in Table 3 for more detail. Of the total children in the study, 24.5% of children were diagnosed with undernutrition, while 75.45% were not diagnosed with undernutrition, indicating that 24.55% of children were stunted, underweight, or wasted. This implies that the prevalence of total undernutrition in children is about 24.55% in Gambia (see Table 3).

**Table 3:** Cross classification of undernutrition indicators and resultant frequency distribution

Undernourished indicators	Frequency (%)
Non- undernourished	1810 (75.45)
Stunting only	119 (4.96)
Underweight only	157 (6.54)
Wasting only	22 (0.92)
Stunting and underweight	171 (7.13)
Stunting and wasting	0 (0.0)
Underweight and wasting	86 (3.58)
Stunting, underweight and wasting	34 (1.42)

Table 4 depicts all possible pairwise dependencies between three indicators of malnutrition using an odds ratio (OR). The odds ratios for wasting and stunting, wasting and underweight, and underweight and stunting were 1.75 (95% CI = 1.48–2.07), 46.54 (95% CI = 28.98–77.85), and 16.74 (95% CI = 12.63–22.30), respectively. One is not included in the dependency’s 95% confidence interval. This indicates a dependency between the indicators of undernutrition, and thus fitting a multivariate binary logistic model for the three indicators is appropriate to account for their dependency and measure the effects of the predictors. Therefore, Table 6 shows a multivariate binary logistic regression analysis of stunting, underweight, and wasting given the other predictors.

**Table 4:** Pairwise dependency between undernutrition indicators using odds ratio (OR)

	Stunting	Underweight
Wasting	OR (95% CI))	OR (95% CI))
	1.75 (1.48, 2.07)	46.54 (28.98, 77.85)
Underweight	16.74 (12.63, 22.30))	

Table 5 shows a bivariable analysis of the relationship between predictors and each indicator of undernutrition. Mothers’ anemia level, number of under-five children in the household, birth type of children, size of children, age of children, mother’s body mass index, and wealth index were independent predictors of stunted, underweight, and wasted children (*p*-value less than 0.05).

**Table 5:** Bivariate Analysis of stunting, underweight, and wasting with the predictors

Predictors	Stunting		Underweight		Wasting	
	Chi-square	*P*-value	Chi-square	*P*-value	Chi-square	*P*-value
Region	43.089	< 0.001	13.252	0.066	8.615	0.281
Residence	16.551	< 0.001	2.873	0.090	1.026	0.311
Mother education level	7.392	0.025	0.319	0.852	2.065	0.356
Source of drinking water	0.448	0.503	0.378	0.539	2.095	0.088
Religion	1.448	0.229	4.709	0.030	1.149	0.284
Ethnicity	13.815	0.182	7.315	0.695	5.364	0.066
Number of household members	9.799	0.007	4.633	0.099	1.847	0.397
Sex of household head	2.222	0.136	0.061	0.806	0.001	0.999
Husband education level	11.576	0.003	8.577	0.014	2.583	0.275
Breastfeeding	0.725	0.395	0.010	0.921	0.556	0.456
Wealth index	38.104	< 0.001	11.431	0.022	6.545	0.162
Body mass index of mother	20.604	< 0.001	40.860	< 0.001	12.497	0.002
Sex of children	1.050	0.305	0.076	0.782	3.988	0.046
Age of children	31.866	< 0.001	30.670	< 0.001	11.746	0.019
Diarrhea	8.225	0.004	7.467	0.006	1.445	0.229
Fever	0.433	0.511	4.028	0.045	2.534	0.111
Cough	0.255	0.614	0.046	0.830	0.117	0.732
Size of children at birth	31.878	< 0.001	114.296	< 0.001	41.716	< 0.001
Birth type of children	35.401	< 0.001	22.566	< 0.001	0.674	0.412
Birth order of children	17.314	0.003	1.740	0.628	1.009	0.799
Number of under-five children in the household	11.943	0.003	10.336	0.006	2.577	0.276
Mother anemia level	4.600	0.032	9.354	0.002	5.556	0.018
Children anemia level	41.316	< 0.001	27.283	< 0.001	0.096	0.757

### Parameter estimation

The predictors included in this study: children’s birth type, anemia level, and age were common determinants that were significantly associated with stunting and underweight. The size of children at birth was found to be significantly related to stunting, underweight, and wasting. Furthermore, the mother’s body mass index was a predictor that was only significantly associated with stunting. The estimated odds of a child with multiple births being stunted and underweight were 1.153 and 1.159 times higher than the estimated odds of a child with a single birth, respectively. This suggests that a child born to a single birth is less likely to be stunted and underweight than a child born to multiple births. The estimated odds of an anemic child being stunted and underweight were 1.022 and 1.033 times higher than the estimated odds of a non-anemic child, respectively. This refers to the estimated odds of the anemic child being stunted, and underweight was lower by 2.2 and 3.3% of the estimated odds of a non-anemic child. On the other hand, an anemic child was more likely to be stunted and underweight compared to a non-anemic child. The estimated odds of children whose age was 12 to 23 months being stunted and underweight were 1.041 and 1.071 times higher than the estimated odds of children whose age was 0 to 11 months, respectively. This indicates that children whose age was 12 to 23 months were more likely to be stunted and underweight as compared to children whose age was between 0 and 11 months. Small-born children had a higher risk of being stunted, underweight, and wasted than normal-born children. This implies that a child of a small birth size is more likely to be stunted, underweight, and wasted than a child of an average birth size. Children from normal and overweight mothers had 0.916 and 0.890 times the odds of being stunted, respectively, as did children from thin mothers. This means that children born to normal or overweight mothers were less likely to be stunted than children born to thin mothers (see Table 6).

**Table 6:** Parameter estimation of undernutrition indicators using Multivariate logistic regression

Predictors	Stunting		Underweight		Wasting	
	AOR (95% CI)	*P*-value	AOR (95%CI)	*P*-value	AOR (95%CI)	*P*-value
Intercept	2.888 (2.604, 3.203)	< 0.001	0.150 (0.146, 0.155)	< 0.001	3.376 (2.958, 3.852)	< 0.001
Region						
Banjul	Ref.		Ref.		Ref.	
Kanifing	0.999 (0.945, 1.055)	0.966	1.012 (0.944, 1.085)	0.741	1.002 (0.981, 1.022)	0.882
Brikama	1.009 (0.957, 1.063)	0.745	1.000 (0.935, 1.069)	0.990	1.002 (0.982, 1.022)	0.843
Mansakanko	0.997 (0.939, 1.060)	0.932	0.995 (0.921, 1.075)	0.987	1.002 (0.979, 1.025)	0.867
Kerewan	1.006 (0.950, 1.066)	0.832	1.002 (0.931, 1.079)	0.959	1.005 (0.983, 1.027)	0.649
Kuntaur	1.022 (0.961, 1.086)	0.500	0.983 (0.909, 1.062)	0.659	1.000 (0.977, 1.023)	0.983
Janjanbureh	1.020 (0.961, 1.082)	0.522	1.008 (0.934, 1.087)	0.844	1.004 (0.982, 1.027)	0.711
Basse	1.039 (0.980, 1.102)	0.196	1.024 (0.951, 1.103)	0.527	1.002 (0.980, 1.024)	0.874
Residence						
Urban	Ref.		Ref.		Ref.	
Rural	0.987 (0.953, 1.022)	0.463	0.981 (0.939, 1.026)	0.404	1.000 (0.987, 1.013)	0.989
Mother education level						
No education	0.999 (0.971, 1.027)	0.918	1.009 (0.974, 1.045)	0.626	1.002 (0.992, 1.013)	0.706
Primary	1.008 (0.980, 1.037)	0.571	1.033 (0.997, 1.070)	0.074	1.001 (0.991, 1.012)	0.833
Secondary +	Ref.		Ref.		Ref.	
Source of drinking water						
Unimproved	1.006 (0.965, 1.049)	0.777	1.026 (0.973, 1.082)	0.351	1.004 (0.988, 1.019)	0.662
Improved	Ref.		Ref.		Ref.	
Religion						
Islam	Ref.		Ref.		Ref.	
Christianity	0.986 (0.854, 1.138)	0.849	0.929 (0.774, 1.116)	0.432	0.998 (0.945, 1.053)	0.933
Number of households						
Small	Ref.		Ref.		Ref.	
Medium	1.010 (0.962, 1.060)	0.699	1.014 (0.952, 1.079)	0.671	0.999 (0.980, 1.017)	0.885
Large	1.014 (0.962, 1.068)	0.613	1.012 (0.946, 1.082)	0.726	0.998 (0.978, 1.018)	0.840
Ethnicity						
Mandinka	Ref.		Ref.		Ref.	
Wollof	1.007 (0.974, 1.042)	0.667	1.012 (0.969, 1.056)	0.599	1.002 (0.989, 1.015)	0.798
Jola/Karoninka	1.041 (0.989, 1.096)	0.121	1.027 (0.962, 1.097)	0.417	1.001 (0.982, 1.021)	0.895
Fula/Tukulur	1.011 (0.982, 1.041)	0.445	1.022 (0.985, 1.061)	0.249	1.001 (0.990, 1.012)	0.917
Serere	1.005 (0.938, 1.077)	0.887	1.062 (0.972, 1.159)	0.183	1.000 (0.975, 1.027)	0.988
Serahuleh	0.981 (0.941, 1.022)	0.350	0.982 (0.931, 1.034)	0.487	0.998 (0.983, 1.014)	0.830
Creole/Aku	1.086 (0.877, 1.345)	0.448	1.095 (0.834, 1.437)	0.515	0.991 (0.915, 1.074)	0.833
Manjago	1.013 (0.825, 1.244)	0.903	1.023 (0.787, 1.328)	0.866	0.992 (0.919, 1.072)	0.846
Bambara	1.014 (0.920, 1.117)	0.756	1.026 (0.907, 1.161)	0.679	0.998 (0.962, 1.035)	0.921
Other	0.969 (0.815, 1.153)	0.726	1.027 (0.823, 1.280)	0.817	1.009 (0.945, 1.077)	0.784
Non-Gambian	0.999 (0.963, 1.036)	0.945	1.006 (0.960, 1.054)	0.806	1.000 (0.986, 1.014)	0.986
Sex of household head						
Male	Ref.		Ref.		Ref.	
Female	1.003 (0.973, 1.034)	0.830	1.008 (0.969, 1.048)	0.698	0.999 (0.988, 1.011)	0.906
Husband education						
No education	Ref.		Ref.		Ref.	
Primary	0.999 (0.971, 1.028)	0.940	0.977 (0.943, 1.013)	0.215	0.998 (0.987, 1.009)	0.715
Secondary +	0.993 (0.964, 1.023)	0.629	0.981 (0.944, 1.019)	0.312	1.001 (0.989, 1.012)	0.915
Breastfeeding						
No	Ref.		Ref.		Ref.	
Yes	0.991 (0.968, 1.015)	0.475	0.980 (0.951, 1.009)	0.1778	0.997 (0.989, 1.006)	0.562
Wealth index						
Poorest	Ref.		Ref.		Ref.	
Poorer	0.988 (0.960, 1.018)	0.441	0.998 (0.961, 1.036)	0.923	1.002 (0.991, 1.013)	0.728
Middle	0.981 (0.944, 1.019)	0.318	0.992 (0.945, 1.041)	0.740	1.003 (0.989, 1.018)	0.654
Richer	0.956 (0.911, 1.003)	0.063	0.952 (0.896, 1.012)	0.1112	1.005 (0.988, 1.024)	0.554
Richest	0.972 (0.921, 1.026)	0.297	0.973 (0.908, 1.042)	0.434	1.001 (0.981, 1.022)	0.892
Body mass index of mother						
Thin	Ref.		Ref.		Ref.	
Normal	0.979 (0.946, 1.014)	0.234	0.916 (0.877, 0.959)	< 0.001	0.994 (0.981, 1.007)	0.386
Overweight	0.964 (0.929, 1.001)	0.058	0.890 (0.849, 0.934)	< 0.001	0.994 (0.980, 1.008)	0.393
Sex of children						
Male	Ref.		Ref.		Ref.	
Female	0.994 (0.974, 1.013)	0.521	0.999 (0.974, 1.024)	0.925	0.997 (0.989, 1.004)	0.421
Age of children in the month						
0 to 11	Ref.		Ref.		Ref.	
12 to 23	1.041 (1.004, 1.079)	0.029	1.071 (1.023, 1.122)	0.003	1.003 (0.989, 1.017)	0.684
24 to 35	1.001 (0.962, 1.042)	0.959	1.023 (0.973, 1.077)	0.373	0.999 (0.984, 1.014)	0.850
36 to 47	1.008 (0.970, 1.048)	0.675	1.014 (0.965, 1.064)	0.588	0.997 (0.983, 1.011)	0.686
48 to 59	0.995 (0.956, 1.036)	0.818	1.008 (0.958, 1.060)	0.768	0.999 (0.984, 1.014)	0.895
Diarrhea prevalence in the last two weeks prior to the survey						
No	Ref.		Ref.		Ref.	
Yes	1.015 (0.989, 1.043)	0.253	1.018 (0.985, 1.053)	0.285	1.000 (0.990, 1.010)	0.959
Fever prevalence in the last two weeks prior to the survey						
No	Ref.		Ref.		Ref.	
Yes	0.999 (0.971, 1.028)	0.941	1.031 (0.994, 1.069)	0.103	1.003 (0.992, 1.014)	0.619
Cough prevalence in the last two weeks prior to the survey						
No	Ref.		Ref.		Ref.	
Yes	1.004 (0.977, 1.032)	0.770	1.001 (0.979, 1.013)	0.228	1.002 (0.988, 1.005)	0.670
Size of children at birth						
Small	Ref.		Ref.		Ref.	
Average	0.965 (0.947, 0.988)	0.002	0.885 (0.852, 0.919)	< 0.001	0.989 (0.978, 0.998)	0.046
Large	0.956 (0.982, 0.985)	0.003	0.838 (0.807, 0.871)	< 0.001	0.992 (0.981, 1.003)	0.158
Birth type of children						
Single	Ref.		Ref.		Ref.	
Multiple	1.153 (1.090, 1.220)	< 0.001	1.159 (1.078, 1.245)	< 0.001	1.000 (0.978, 1.021)	0.931
Birth order of children						
First	1.000 (0.971, 1.031)	0.986	1.081 (0.979, 1.057)	0.375	1.001 (0.990, 1.012)	0.871
Two to three	1.021 (0.988, 1.055)	0.212	1.027 (0.985, 1.070)	0.218	1.000 (0.988, 1.055)	0.998
Four to five	1.024 (0.989, 1.060)	0.175	1.036 (0.992, 1.083)	0.113	1.001 (0.988, 1.014)	0.906
Six and more	Ref.		Ref.		Ref.	
Number of under-five children in the household						
Only one	Ref.		Ref.		Ref.	
Two children	1.000 (0.966, 1.043)	0.968	1.014 (0.972, 1.056)	0.518	1.001 (0.989, 1.014)	0.851
3 and more	1.009 (0.972, 1.047)	0.643	1.034 (0.986, 1.084)	0.173	1.004 (0.990, 1.018)	0.598
Children anemia level						
Not anemic	Ref.		Ref.		Ref.	
Anemic	1.022 (1.001, 1.045)	0.044	1.033 (1.005, 1.062)	0.020	1.001 (0.991, 1.007)	0.759
**Dependency**		**Odds ratio (OR)**		**P-value**		
Stunting/Underweight		15.87		< 0.001		
Stunting/Wasting		0.995		0.071		
Underweight/Wasting		46.34		< 0.001		

Based on the estimation of undernutrition indicators using a multivariate binary logistic regression model, concordant and discordant proportions were calculated. 81.6% of stunted, underweight, or wasting children have a good chance of predicting their stunting, underweight, or wasting levels. The concordant proportion was very high in this regard. This shows that the model’s ability to explain the relationship between the indices was adequate and fitted the data well.

## Discussion

The relationship between undernutrition indicators such as stunting, underweight, and wasting for under-five children and other predictors was discussed concisely in this study, and the effects of predictors were assessed using data from the 2019/20 Gambian Demographic Health Survey (GDHS). A multivariate binary logistic regression model was used to determine the effect of predictors on undernutrition indicators such as wasting, underweight, and stunting. The pairwise dependency between undernutrition indicators was confirmed in this model given other predictors, and model parameter estimates for the significant predictors (at a 5% significance level) are presented and interpreted. This study reported that underweight is significantly associated with both stunting and wasting, and the study also revealed that underweight is a composite measure of stunting and wasting. This result is in line with the studies in Ethiopia, India, and Malawi [43, 45] respectively. However, in the previous study, when the association between the three indicators was assessed, the effect of other predictors was not taken into consideration. Nevertheless, in this study, when the relationship between the three indicators was assessed, the consequence of other children, community, maternal, and household factors was not taken into consideration. On the other hand, there was an insignificant association between stunting and wasting, which is in line with a study on Ghanaian preschool children and a study in Uganda on concurrently wasted and stunted children aged 6–59 months [40, 49], which suggested that the prevalence of concurrent stunting and wasting among under-five children was low.

The prevalence of stunting and wasting for under-five children in the current study was lower than the world cases of 22 and 6.7%, respectively, but the prevalence of underweight is higher according to the 2019/20 GDHS report [37]. This indicates that stunting and wasting prevalence have decreased, whereas the prevalence of underweight has increased in Gambia. This study coincides with the studies [50–52] in Ethiopia, Pakistan, and Ghana, respectively. The possible reasons are sample size and geographical differences [53].

In this finding, anemia level of children, age of children, and birth type of children were statistically significant determinants of both stunting and underweight. The current study is consistent with the studies in [50–52] Ethiopia, Pakistan, and Ghana, respectively. Anemic children were more likely to be stunted and underweight as compared to non-anemic children. This study is in line with the previous findings [35, 51]. The possible justification is that poor food status is associated with poor health and, consequently, pollution and invasions also have synergistic consequences of micronutrient deficiencies for the incidence of anemia. Also, malnourished kids are vulnerable to micronutrient shortages like iron, vitamin A, vitamin B12, and folic acid, which are helpful for hemoglobin and DNA fusion during the period of red blood cell production and, in turn, consequences of anemia [54]. Anemia and malnutrition frequently share common causes; it is likely that many types of malnutrition will coexist in similar children and contribute to the development of anemia in a synergistic manner. Furthermore, in malnourished children, the intestinal epithelium may suffer, weakening absorption and contributing to the extension and fall of anemia [55]. Therefore, low hemoglobin levels may also interfere with the direct development of childhood.

Our finding indicated that children whose age was between 12 and 23 months were more likely to be stunted and underweight as compared to children aged 0 to 11 months. This study is consistent with the previous studies done in Ethiopia, Burkina Faso, and Bangladesh [22, 56, 57], which found that the danger of malnutrition increased along with the increase in the age of a child. This is attributable to the late introduction of complementary foods with low food quality [58]. Similarly, the risk of child malnutrition with increasing child age pressures the need for good and suitable commencement of additional feeding to meet the rising nutritional requirements of children [51]. In this study, the size of children at birth was an important determinant of stunting, underweight, and wasting. The risk of children being stunted, underweight, and wasted was higher for small-born children as compared to normal-born children. This result is in line with the studies in Pakistan [56], Ethiopia, Burkina Faso, and Nepal [4, 51, 59]. Perceived child size at birth had a significant impact on the child’s nutritional status [60], as low birth size is a marker of controlled intrauterine development [61].

This finding indicated that children who had multiple types of birth were more likely to be stunted and underweight as compared to children who had a single birth type. This study is consistent with the studies [20, 29, 56]. This might be due to children who are twins might not get exclusive breast milk at early ages, and this reduces their resistance and disposition to diarrhea. Similarly, the quality of attention from parents is reduced. So they are easily disposed to diverse diseases and malnutrition. The body mass index of a mother was the most important determinant of a child’s stunting. Children from normal and overweight mothers were less likely to be stunted as compared to children from thin mothers. This study coincides with the studies [9, 29, 56, 62, 63]. As maternal body mass index is a significant factor of child undernutrition and is influenced by maternal nutrition, in order to develop child growth, an appropriate diet is vital for mothers during the prenatal and postnatal periods. Healer mothers are less likely to have malnourished children [29].

One of the study’s strengths was the use of a nationally representative sample and objectively measured biomarkers such as hemoglobin level, weight, and height. When using binary or ordinal logistic regression, the association between stunting, underweight, and wasting is ignored. Using multivariate logistic regression may be a better option in this case. This statistical model is used to simulate two or more binary outcome variables at the same time and assess their relationship in relation to other predictors. It meets the criteria for modeling marginal likelihood as a function of explanatory variables. Simultaneously, the model investigates the relationship between stunting, underweight, and wasting in children under the age of five. The inclusion of various explanatory variables (child, maternal, community, and household variables) may have improved the study’s comprehensiveness and allowed for adjustment for various potential confounding variables. This study, however, has significant limitations. First, the study’s cross-sectional design precludes establishing temporal relationships and making causal inferences. Second, data collection on some variables based on respondents’ memories of past events may have introduced recall bias. Third Because this study relied on secondary data, we were unable to investigate all potential contributors to childhood malnutrition, such as eating habits, parasite infestations, nutritional supplement use, and gestational birth weight. A prospective study focusing on more specific and relevant variables would yield more useful data. In conclusion, as the prevalence of undernutrition indicators such as stunting, underweight, and wasting is difficult for children’s development, there is a need for policymakers and stakeholders to direct resources to reduce numerous impacts due to undernutrition by taking into consideration the important determinants that were identified by this study.

## Conclusion

The prevalence of stunting and wasting for under-five children in Gambia was lower than the world prevalence, but the prevalence of being underweight was higher. Children who are underweight have a significant association with both stunting and wasting. However, there was a lack of association between stunting and wasting. The age of the child, the child’s anemia level, and the birth type of the child are the common important determinants of stunting and underweight. The small birth size of a child was highly associated with a higher risk of stunting, underweight, and wasting among under five-year-olds. A child born to a normal mother had a lower chance of being stunted than a child born to a thin mother. The authors would like to recommend that governmental and non-governmental stakeholders develop short- and long-term food supplementation programs that allow them to reduce the prevalence of child malnutrition and its associated health effects by taking into account the important determinants. Furthermore, it is preferable to strengthen strategies for early detection and management of children’s anemia in order to reduce the prevalence of childhood stunting, wasting, and underweight.

## Data Availability

The data used in this article were available on http://dhsprogram.com.

## Abbreviations

DHS: Demographic health survey
EAs: Enumeration areas
GDHS: Gambian demographic and health survey
Ref.: Reference Category HAZ Height for age standardized score;
WAZ: Weight for age standardized score
WHZ: Weight for height standardized score
OR: Odds ratio
